# Resting State BOLD Variability of the Posterior Medial Temporal Lobe Correlates with Cognitive Performance in Older Adults with and without Risk for Cognitive Decline

**DOI:** 10.1523/ENEURO.0290-19.2020

**Published:** 2020-05-20

**Authors:** Tyler J. Good, Joshua Villafuerte, Jennifer D. Ryan, Cheryl L. Grady, Morgan D. Barense

**Affiliations:** 1 Rotman Research Institute, Baycrest Health Sciences, Toronto M6A 2E1, Ontario; 2 Department of Psychology, University of Toronto, Toronto M5S 3G3, Ontario; 3 Department of Psychiatry, University of Toronto, Toronto M5T 1R8, Ontario

**Keywords:** aging, BOLD fMRI, BOLD variability, MCI

## Abstract

Local brain signal variability [SD of the BOLD signal (SD_BOLD_]] correlates with age and cognitive performance, and recently differentiated Alzheimer’s disease (AD) patients from healthy controls. However, it is unknown whether changes to SD_BOLD_ precede diagnosis of AD or mild cognitive impairment. We compared ostensibly healthy older adult humans who scored below the recommended threshold on the Montreal cognitive assessment (MoCA) and who showed reduced medial temporal lobe (MTL) volume in a previous study (“at-risk” group, *n* = 20), with healthy older adults who scored within the normal range on the MoCA (“control” group, *n* = 20). Using multivariate partial least-squares analysis we assessed the correlations between SD_BOLD_ and age, MoCA score, global fractional anisotropy, global mean diffusivity, and four cognitive factors. Greater SD_BOLD_ in the MTL and occipital cortex positively correlated with performance on cognitive control/speed tasks but negatively correlated with memory scores in the control group. These relations were weaker in the at-risk group. A *post hoc* analysis assessed associations between MTL volumes and SD_BOLD_ in both groups. This revealed a negative correlation, most robust in the at-risk group, between MTL SD_BOLD_ and MTL subregion volumetry, particularly the entorhinal and parahippocampal regions. Together, these results suggest that the association between SD_BOLD_ and cognition differs between the at-risk and control groups, which may be because of lower MTL volumes in the at-risk group. Our data indicate relations between MTL SD_BOLD_ and cognition may be helpful in understanding brain differences in individuals who may be at risk for further cognitive decline.

## Significance Statement

Moment-to-moment variability in the BOLD signal, once dismissed as nuisance noise, is now understood to be an information-bearing signal. BOLD variability correlates with age and cognitive performance and was recently used to differentiate Alzheimer’s disease (AD) patients from controls. As AD is a progressive disease, AD patients may benefit from its early detection. We found that older adults at-risk for cognitive decline showed differences in the relationships between BOLD variability and cognitive performance, relative to healthy controls. Notably, the differences were strongest in medial temporal lobe (MTL), areas where AD is known to begin. Our data suggest that correlations between MTL BOLD variability and cognition may be useful for understanding brain differences in individuals at risk for further cognitive decline.

## Introduction

Alzheimer’s disease (AD) is characterized by cognitive decline, especially to memory performance (Alzheimer’s Association, 2017). Epidemiological evidence suggests age is the strongest risk factor for AD (Alzheimer’s Association, 2017) and because of its progressive nature, patients may benefit from its early detection. Many studies have tried to predict AD in preclinical patients (Jack and Holtzman, 2013; Jack et al., 2013; Dubois et al., 2016), but efforts are ongoing.

Temporal variability of the BOLD signal (SD_BOLD_) correlates with age (Garrett et al., 2010) and cognitive performance (Garrett et al., 2013a, 2011). Initially, older age and poorer cognitive performance were primarily associated with lower SD_BOLD_ during task-based fMRI and therefore SD_BOLD_ was conceptualized as an indicator of brain health (Garrett et al., 2010, 2011, 2013a; Grady and Garrett, 2014; Guitart-masip et al., 2016). However, early studies of SD_BOLD_ also found age-related increases of variability in the inferior temporal gyrus (Garrett et al., 2010), hippocampus (Garrett et al., 2011), and other areas. More recently, studies have corroborated these findings, showing aging is related to greater resting-state variability in some networks, but lower variability in others (Nomi et al., 2017). Another study found some cognitive domains were positively associated with resting-state SD_BOLD_, whereas others were inversely related (Burzynska et al., 2015). Furthermore, in clinical populations, stroke was associated with resting-state variability increases in the left postcentral gyrus (Kielar et al., 2016); multiple sclerosis with resting-state increases in temporal gyrus and dorsal medial prefrontal cortex (Petracca et al., 2017); 22q11.2 deletion syndrome with resting-state increases in temporal lobe and caudate (Zöller et al., 2017); medial temporal lobe epilepsy with task-based increases in various cortical and subcortical regions (Protzner et al., 2013); and most notably, AD with resting-state increases in superior frontal gyrus, precentral gyrus, and putamen (Scarapicchia et al., 2018). Together, these findings raise the possibility that SD_BOLD_ may support behavior differently in healthy aging and disease.

Because of its sensitivity to age and cognitive performance, SD_BOLD_ is a promising tool for understanding age-related pathologies like AD. A few studies provide hints regarding what physiological mechanisms may be driving SD_BOLD_. Researchers have demonstrated a relation between age-related changes in SD_BOLD_ and dopaminergic neuromodulation (Garrett et al., 2015). Additionally, higher SD_BOLD_ may reflect lower dimensional functional integration within brain networks (Garrett et al., 2018), suggesting that the optimal (and more variable) brain would have the lowest dimensionality (and therefore more tightly integrated networks) necessary given the contextual demands. This framework may explain some of the SD_BOLD_ findings across the literature. Notably, Burzynska et al. (2015) found that resting-state SD_BOLD_ was positively correlated with complex tasks that required greater dynamic range, but negatively associated with less complex tasks. Further, increased local variability may be common across numerous clinical populations (Protzner et al., 2013; Kielar et al., 2016; Petracca et al., 2017; Zöller et al., 2017; Scarapicchia et al., 2018) because of a shift toward suboptimal dynamics in the face of structural brain damage. Similarly, increased integration between brain networks with older age is a robust finding in healthy adults (Damoiseaux, 2017). Regardless, if SD_BOLD_ reliably differs in individuals showing early signs of cognitive decline from their cognitively normal counterparts, it may prove useful in understanding the brain differences that may precede mild cognitive impairment (MCI) and AD diagnosis.

The present study compares relations between cognitive performance and SD_BOLD_ in ostensibly normal older adults identified as at-risk of developing MCI and age-matched controls. The at-risk group scored below the threshold Montreal cognitive assessment (MoCA) score (<26), whereas controls scored in the normal range. Although this grouping was based solely on MoCA score, previous investigations of this sample observed differences in other brain and behavioral domains consistent with preclinical cognitive and neural decline (Olsen et al., 2017; Yeung et al., 2017). We hypothesized that the at-risk group would have greater SD_BOLD_ than controls, based on findings in patients with diagnosed AD (Scarapicchia et al., 2018). We also hypothesized that the relationship between SD_BOLD_ and cognition would depend on group membership and cognitive domain. Specifically, we expected SD_BOLD_ to be positively associated with more complex tasks, whereas less complex tasks would be negatively associated with SD_BOLD_, mirroring work by Burzynska et al. (2015). Last, as reductions in medial temporal lobe (MTL) volume were previously noted in our sample (Olsen et al., 2017; Yeung et al., 2017), we conducted a *post hoc* analysis to determine if SD_BOLD_ was associated with MTL volumes in those regions showing group differences in SD_BOLD_ identified in the present study.

## Methods

### Participants

Forty community-dwelling adults were recruited from participant databases at the Rotman Research Institute at Baycrest and the University of Toronto. Participants (Table 1) were chosen from the databases such that the at-risk group (*n* = 20) scored below the cutoff on the MoCA (all <26, *M *=* *23.4, SD = 1.9; M age = 71.5, SD age = 6.1, M education = 15.5, SD education = 2.9, 17 female), whereas the control group (*n* = 20) scored above the cutoff (M = 27.9, SD = 1.7; M age = 70.3, SD age = 4.5, M education = 16.5, SD education = 2.8, 13 female). There were no group differences in age, *t*_(38)_ = 1.3, *p *=* *0.20, or years of education, *t*_(38)_ = −2.2, *p *=* *0.40, but a significant difference in MoCA score, *t*_(38)_ = 7.87, *p *<* *0.001. The sample has been reported on, and described in more detail, in previous papers (Olsen et al., 2017; Yeung et al., 2017). No participants were removed due to head motion (absolute head motion at a single time point was no greater than 2 mm and relative head motion did not exceed 2.5 mm for all participants).

**Table 1:** Demographic characteristics

	At-risk (SD)	Controls (SD)
*n*	20	20
MoCA	23.4 (1.9), all <26	27.9 (1.7), all >26
Age	71.5 (6.5)	70.3 (4.5)
Education	15.5 (2.9)	16.5 (2.8)
Sex	17 female	13 female

### Neuropsychological battery

Participants performed a battery of neuropsychological tests to characterize their cognitive function in a separate session before the MRI scan. The battery included the Rey–Osterrieth complex figure test (RCFT; Osterreith, 1944), Visual Object and Space Perception Battery (VOSP; Warrington and James, 1991), Digit Span subset of the Wechsler Adult Intelligence Scale (WAIS; Wechsler, 1999; Wechsler et al., 2008), Logical Memory subset of the Wechsler Memory Scale (WMS), Ed 4 (Wechsler, 2009), Trail Making Test A and B (Reitan and Wolfson, 1985), and the Wechsler Abbreviated Scale of intelligence (WASI), Ed 4 (Wechsler, 1999). Collectively, the battery assessed intelligence, visuospatial performance, cognitive control/speed, and memory (Table 2) using 14 tests.

**Table 2:** Factor loadings for neuropsychological tests

Neuropsychological Test	Subtest	1. Visuospatial	2. Cognitive control/speed	3. Memory	4. Intelligence
RCFT	Immediate recall	**0.87**	-0.03	0.06	0.13
	Delayed recall	**0.84**	-0.01	0.08	0.24
VOSP	Silhouettes	**0.58**	0.13	0	0.23
	Progressive silhouettes	**0.69**	0.23	-0.09	0.2
WASI	Block design	**0.53**	0.16	0.37	**0.53**
Trail making test (TMT)	Alternating (version A)	**0.46**	**0.54**	0.11	−0.14
	Sequential (version B)	**0.41**	**0.69**	0.3	0.18
WAIS	Digit span forward	−0.31	**0.72**	−0.13	0.18
	Digit span backward	0.08	**0.79**	0.21	0.08
WMS	Immediate recall	−0.08	0.2	**0.85**	0.28
	Delayed recall	0.09	0.05	**0.88**	−0.02
WASI	Matrix reasoning	0.36	0.6	0.06	**0.42**
	Vocabulary	0.24	0.19	0.15	**0.83**
	Similarity	0.24	0.08	0.05	**0.8**
Proportion of variance accounted for by each factor		0.24	0.18	0.15	0.13

*Loadings >|0.40| are bold to assist with factor interpretation.

Principal component analysis (PCA) was used (“principal” function in R) to expose the latent structure within the battery and to reduce the dimensionality of the dataset. Data were transformed if significantly skewed (*p* < 0.05) using square root, or log10 to improve the correlation structure. If necessary, variables were inverted so that higher scores always indicated better performance. Point of inflection on a scree plot was used to identify the number of components to keep. After extracting this number of factors, an oblique rotation was performed using the “principal” function in R. Factor correlations were assessed, and none exceeded 0.32 (greatest was between PC1 and PC4, *r *=* *0.30), indicating that <10% of the variance was shared between factors. In this case, the solution is nearly orthogonal, and an orthogonal rotation is also appropriate (Tabachnick and Fidell, 2007). As such, a four-factor solution using a varimax rotation was performed using “principal” in R. The identified factors were largely multi-factorial and any single description would be unsatisfactory. However, as a high-level description and to allow identification at a glance, we loosely named the factors as (1) visuospatial, (2) cognitive control and speed of processing (cognitive control/speed), (3) intelligence, and (4) memory based on the primary cognitive functions assessed by the tasks that strongly contributed to each (loading >|0.4|; Table 2). Factor scores for each participant represent the degree to which they express the factor; higher factor scores indicate better performance on the tests reliably contributing to the factor. Factor scores were used in subsequent analyses.

### Imaging procedure

All neuroimaging was performed in a single session with a 3T Siemens Trio scanner using a 12-channel head coil. Participants received a T1-weighted, magnetization-prepared, rapid acquisition with gradient echo image (MP-RAGE) whole-brain anatomic scan (TE/TR = 2.63 ms/2000 ms, 160 axial slices perpendicular to the AC–PC line, 256 × 192 acquisition matrix, voxel size = 1 × 1 × 1 mm, FOV = 256 mm). The T1-weighted MP-RAGE scan was used for slice placement during the acquisition of a subsequent high-resolution T2-weighted scan in an oblique-coronal plane, perpendicular to the hippocampal long axis (TE/TR = 68 ms/3000 ms, 20–28 slices depending on head size, 512 × 512 acquisition matrix, voxel size = 0.43 × 0.43 × 3 mm, no skip, FOV = 220 mm).

The DTI duration was 9:22 (min:s). There were 68 slices taken perpendicular to the AC–PC line with the following parameters: 34 directions, TE/TR = 84 ms/7900 ms, FOV = 242 mm, flip angle 90°, *b* value = 1000 s/mm^2^, voxel size = 2.2 × 2.2 × 2.2 mm.

Participants were instructed to keep their eyes open and focused on a fixation cross during the resting-state scan. It consisted of 180 BOLD-sensitive slices (6 min, 180 time points, TR = 2000 ms, TE = 30 ms, flip angle = 70°, FOV = 200 mm, 30 slices, 64 × 64 matrix, voxel size = 4.0 × 4.0 × 4.0 mm).

### DTI preprocessing

Eddy current-induced distortions were corrected using FSL’s “eddy_correct” command, and the diffusion gradient vectors rotated accordingly. The MNI152_T1_1mm standard brain was then registered to subject T1-*w* space using a nonlinear registration conducted with Advanced Normalization Tools (ANTs). Warps produced in this step were used to transform MNI AAL cortical masks using ANTs’ “WarpImageMultiTransform” executable and a nearest neighbor interpolation. FSL’s FLIRT function was used to register subject T1-*w* images to DTI space. Diffusion tensor models were fitted at each voxel by FSL’s “dtifit”.

### fMRI preprocessing

Resting-state fMRI preprocessing was done using FMRIB’s FEAT toolbox. The following steps were performed: (1) brain extraction with “bet” (Smith, 2002), (2) motion correction with MCFLIRT (Jenkinson et al., 2002), (2) slice timing correction, (3) spatial smoothing (6 mm), and (4) registration to anatomic volume using FLIRT (Jenkinson et al., 2002). Next, a bandpass filter was applied (0.01–0.1 Hz). Linear and quadratic detrending was performed, and then all functional volumes were examined for artefacts using independent component analysis (ICA) within-run, within-person, as implemented by FSL/MELODIC (Beckmann and Smith, 2004). Extra de-noising using ICA was performed in light of previous research showing group differences in SD_BOLD_ were enhanced following the procedure (Garrett et al., 2010). Noise components were identified using the following criteria, by two independent coders: (1) spiking (components dominated by abrupt time series spikes ∼≥6 SD), (2) motion (prominent edge or “ringing” effects, sometimes, but not always accompanied by large time series spikes), (3) susceptibility and flow artefacts (prominent air-tissue boundary or sinus activation, typically represents cardio/respiratory effects), (4) white matter and/or ventricle activation, (5) low-frequency signal drift, (6) high-power in high-frequency ranges unlikely to represent neural activity (∼≥75% of total spectral power present ∼>0.13 Hz), and (7) spatial distribution [“spotty”, or “speckled” spatial pattern that appears scattered randomly across ∼≥25% of the brain, with few if any clusters of ∼≥10 contiguous voxels (at 4 × 4 × 4 mm voxel size)]. Generally, decision criteria were applied conservatively, so if there was difficulty classifying a component due to “signal” and “noise” both being present, the component was kept. Components identified as artefacts were regressed out of their respective scan using the “regfilt” function in FSL.

### Voxelwise diffusion tensor imaging analysis

Voxelwise analysis of the fractional anisotropy and mean diffusivity maps was conducted using tract based spatial statistics (TBSS) with FSL’s Diffusion Toolkit (Smith et al., 2006). Briefly, this involves ensuring all subjects’ fractional anisotropy (FA) and mean diffusivity (MD) images were in common [Montreal Neurologic Institute (MNI)] space, using FMRIB’s nonlinear registration tool, FNIRT. A group mean FA and MD image was created, and thinned to produce a mean FA/MD skeleton that represents the centers of all tracts common to the group. Each subject’s FA/MD images were then projected onto this skeleton. Nonparametric permutation-based statistics were used to explore differences in FA and MD between groups using FMRIB’s randomize function with 5000 unique permutations. Correction for multiple comparisons was performed using the threshold-free cluster enhancement tool in randomize with threshold *p *<* *0.05. Group comparison PLS could also be used to assess group differences in FA/MD; however, we chose to stay within the FSL/TBSS framework, which was used to generate the FA/MD maps. To get a summary metric of overall white matter integrity, global FA and MD were calculated for each participant. These were simply the averages of all non-zero voxels in the skeletonized FA and MD images.

### BOLD signal variability calculation

To calculate SD_BOLD_, voxelwise time series were extracted from the preprocessed resting fMRI images of each subject and normalized such that the mean across the brain was 100. The mean was then subtracted from each subject’s voxelwise time series, so the data were expressed as deviation from the mean. Voxelwise SD_BOLD_ was then calculated on these time series for each subject. We used code adapted from a previous study (https://github.com/stefanschmidt/vartbx/; Garrett et al., 2013a), simplified to account for our data having only a single condition (resting-state). To restrict our analyses to gray matter, we masked the SD_BOLD_ maps with the gray matter tissue prior provided by FSL thresholded at a probability that a given voxel is gray matter > 0.43. We assessed head motion during the fMRI resting state scan and found our groups did not differ in mean absolute head motion, *t*_(38)_ = −1.03, *p *=* *0.31, or in mean relative head motion, *t*_(38)_ = −0.23, *p *=* *0.82.

### Manual segmentation of MTL subregions

A single rater who was blind to MoCA score/group status performed manual segmentation of three hippocampal (HC) subfields (CA1, dentate gyrus/CA2 and CA3, and subiculum) and four MTL cortices [anterolateral entorhinal cortex (alERC), posteromedial ERC (pmERC), perirhinal cortex (PRC), and parahippocampal cortex (PHC)] using the coronal slices of the T2-weighted images (in-plane resolution: 0.43 × 0.43 mm, 3 mm between slices) in FSLview v3.1. A second rater who was also blind to MoCA score/group status, segmented the same regions to provide an index of inter-rater reliability. The segmentation protocol was largely similar to the Olsen–Amaral–Palombo protocol used for previous volumetric investigations of the MTL (Olsen et al., 2009, 2013; Palombo et al., 2013; Yushkevich et al., 2015). The volumetric analysis of our sample has been described extensively in two previous publications (Olsen et al., 2017; Yeung et al., 2017) and therefore is not outlined further here. The at-risk group showed significantly lower alERC volume than the controls and trending lower volume in the CA1 subfield and PRC, after correction for age (Olsen et al., 2017). We also note that neither absolute nor relative head motion during the resting state scan correlated with any of the seven MTL volumes, *p *>* *0.05.

### Partial least-squares analysis

Partial least-squares (PLS) analysis relates two sets of variables, by identifying linear combinations of variables in both sets that maximally covary together (McIntosh and Lobaugh, 2004; McIntosh and Mišić, 2013). In this study, we used two group comparison PLS analyses. The first related group membership to neuropsychological factor score, whereas the second related voxelwise SD_BOLD_ to group membership. These analyses aimed to find a pattern of neuropsychological factors that differed between groups, or a pattern of brain regions where SD_BOLD_ differed between groups. We also used behavioral PLS to find patterns of SD_BOLD_ that related similarly and differently across groups to several potentially related variables (age, MoCA score, global FA/MD, and scores on our four neuropsychological factors). We included FA and MD in this analysis as a previous study found global FA was strongly associated with the relationship between neuropsychological performance and SD_BOLD_ in a sample of healthy adults a similar age to ours (Burzynska et al., 2015). As such, we had reason to believe FA/MD may scale with SD_BOLD_ regardless of a significant difference in white matter integrity between groups. We also used behavioral PLS to assess associations between neuropsychological test scores and age in each group. This analysis allowed use to check for differential cross-sectional changes with age in terms of cognitive performance.

For the group comparison PLS analyses, a data matrix, X, was composed of scores on the neuropsychological factors, or a vectorized version of the SD_BOLD_ values for all voxels within our whole-brain gray matter mask. These matrices were constructed such that observations (participants nested in groups) corresponded to rows, whereas voxels related to columns. Within-group column means were calculated, and the data in *X* were mean-centered creating M_dev_. The mean-centered data were then subjected to singular value decomposition (SVD): [U, S, V] = SVD(M_dev_), such that USV’ = M_dev_. The resulting latent variables (LVs) consist of left singular vector U, right singular vector V, and the diagonal matrix S. The left singular vector U contains the element saliences (weights) that identify the neuropsychological factors or voxels that make the greatest contribution to the contrast captured by the latent variable. The right singular vector V contains the design salience, which signifies the contribution of each group to the pattern of neuropsychological factors, or voxelwise SD_BOLD_ identified by the latent variable. The scalar singular value, from the diagonal matrix S, denotes the covariance between the data blocks, and indicates strength of the relationship identified by the latent variable.

For the first behavioral PLS, the construction of the input matrix differed from the group comparison PLS, such that it contained the correlations between SD_BOLD_ and age, MoCA score, global FA/MD, and our four cognitive factors for each group. For the second behavioral PLS, the input matrix contained correlations between age and scores on the neuropsychological tests for each group. First, correlations were calculated between the submatrices *X*_nxp_ (*n* participants by *p* voxels or *n* participants by *p* variables), and *Y*_nxb_ (*n* participants by *b* related variables). The correlations were then stacked by group, which created the input matrix subjected to SVD. Behavioral saliences *V*(i) indicate the degree to which brain–behavior relationships are expressed for each variable in *Y*, whereas element saliences *U*(i) indicate the degree to which voxels express these brain–behavior correlations.

Permutation testing was used to determine significance of each latent variable. Rows of the input matrix are randomly reordered and the decompositions performed, as described above. This was done 1000 times, creating a distribution of singular values. Bootstrapping was used to estimate the reliability of individual weights. Participants were randomly resampled (rows in *X*) with replacement while respecting group membership. The resampled matrices were decomposed, as described above. This was done 1000 times, generating a sampling distribution for the weights in the singular vectors. SE was calculated from this sampling distribution, reflecting the stability of the weight. A bootstrap ratio was then calculated for each voxel, by dividing the weight from the singular vector by its bootstrap-estimated SE, and is akin to a *z* score. Confidence intervals were calculated around design/behavior weights using the percentiles derived from the sampling distribution. Brain scores from the behavioral PLS represent the degree to which each subject expresses the contrast identified by the latent variable.

### *Post hoc* comparison of MTL volumetry and MTL SD_BOLD_

A previous study of our sample found the at-risk group had significantly reduced alERC volume relative to controls and trending lower volume in the CA1 subfield and PRC (Olsen et al., 2017). To assess the relation between MTL volumes and SD_BOLD_ in these same regions, we conducted a *post hoc* behavioral PLS. The PLS model compared volume of three HC subfields (CA1, dentate gyrus/CA2 and 3, and subiculum) and four MTL cortices (alERC, pmERC, PRC, and PHC) with SD_BOLD_ in these MTL regions. Specifically, the SD_BOLD_ data were calculated from all voxels in our whole-brain gray matter mask that overlapped with the left/right hippocampal and parahippocampal regions of the automated anatomical labeling (AAL) parcellation (Tzourio-Mazoyer et al., 2002). Critically, this included all regions quantified by volumetry. This SD_BOLD_ data formed the first input, *X*_nxp_, structured *n* participants by *p* voxels. The second input matrix, *Y*_nxr_, contained MTL volume data for each subject, *n*, by and brain region, r. Input matrices *X*_nxp_ and *Y*_nxr_ were subjected to behavioral PLS, as described in the previous section. Brain scores from this PLS represent the degree to which each subject expresses the contrast identified by the latent variable. Similarity of brain scores from the PLS of MTL variability and volumes and the previous behavioral PLS of whole-brain variability and potentially related variables was assessed with Pearson correlation. This showed the degree of association between the pattern of SD_BOLD_ related to the behavioral variables and the pattern of SD_BOLD_ related to MTL volumes.

## Results

### No group differences on brain variables, cognitive measures

Before probing the relations among our variables, we checked for differences in SD_BOLD_, FA, and MD between the at-risk group and controls. A group comparison PLS revealed no significant difference in voxelwise whole-brain gray matter SD_BOLD_ between those at-risk for MCI and controls (*p *>* *0.05). Non-parametric permutation-based testing using FSL’s “randomise” found no significant group differences in voxelwise FA or MD (*p *>* *0.05). Similar to this finding with randomize, group comparison PLS of voxelwise FA and MD showed no group differences (FA, *p *=* *0.68; MD, *p *=* *0.17).

**Figure 1.**
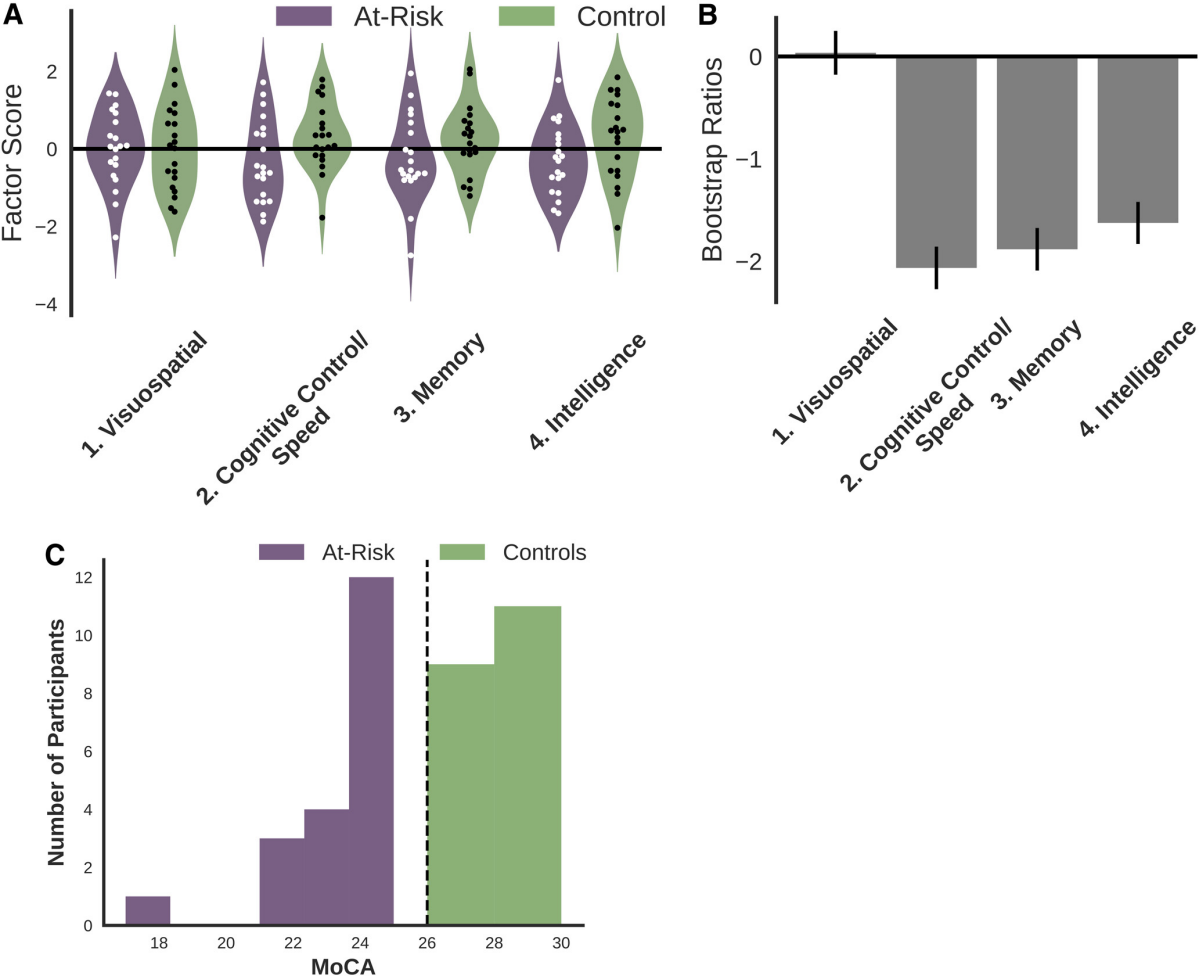
The at-risk group scored lower than controls on three neuropsychological factors (cognitive control/speed, memory, and intelligence), though the group difference did not quite reach significance. ***A***, Violin plots showing the distribution of factor scores for each group. ***B***, Results from a group comparison PLS that found a marginal effect such that the at-risk group scored lower than controls on the cognitive control, memory and intelligence factors (*p *=* *0.053). Bootstrap ratios (BSRs) are plotted and represent a linear combination of the factors weighted by how strongly they contribute to the latent variable. Negative BSRs indicate the at-risk group had lower scores than controls on these factors. BSRs may be interpreted similar to *z* score (>|2.5| akin to *p *<* *0.05), suggesting again that the at-risk group’s lower scores on the control/speed, memory, and intelligence factors was approaching significance. Error bars represent 1 SE = standard error. ***C***, Distribution of MoCA scores in the present sample. The black dashed line indicates the recommended MoCA cutoff score (26 points out of 30).

**Figure 2.**
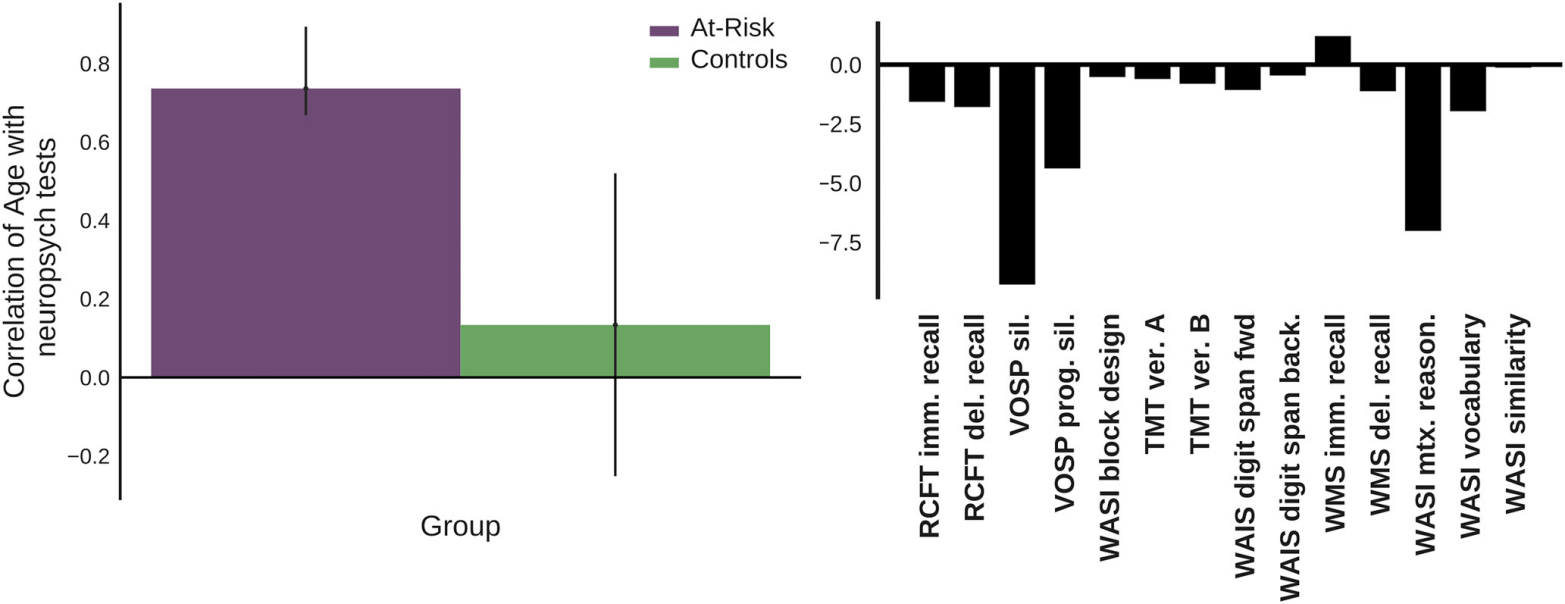
A behavioral PLS (*p *=* *0.01, 87% covariance) found age was negatively correlated with scores on the VOSP silhouette, VOSP progressive silhouette, and WASI matrix reasoning tests in the at-risk group, but not controls. The left bar graph shows the correlation between age and the pattern of neuropsychological tests shown on the right. The error bars represent 95% confidence intervals. Bootstrap ratios are pictured on the right, representing a linear combination of the neuropsychological tests weighted by how much they contribute to the latent variable. Bootstrap ratios may be interpreted like *z* scores, such that bootstrap ratios >|2.5| are considered to be reliably correlated with age in the group(s) that are significantly contributing to the latent variable. Immediate (imm), delayed (del), sillhoutte (sil), version (Ver).

Our PCA of the neuropsychological battery revealed four significant factors, which we designated as follows: (1) visuospatial, (2) cognitive control/speed, (3) memory, and (4) intelligence (Table 2). The groups did not significantly differ in their performance on any of the factors; however, a group comparison PLS showed the at-risk group trended toward lower scores on the control/speed, memory, and intelligence factors (*p *=* *0.053). We also note that the visuospatial factor correlated moderately with age, *r*_(38)_=−0.36, *p *=* *0.02; Fig. 1, though the other factors did not (*p *>* *0.05). For group differences on the individual neuropsychological tests please refer to Table 2 in an earlier paper: Olsen et al., 2017. Briefly, the at-risk group had significantly lower WMS recognition accuracy and WASI matrix reasoning scores (*p *<* *0.01), lower scores on the WMS delayed recall, digit span backward, Trails B, VOSP incomplete letters and number locations (*p *<* *0.05), and trending lower scores on the digit span forward, WASI vocabulary, WASI similarity, and VOSP position discrimination (*p *<* *0.1). A behavioral PLS (Fig. 2) found age was associated with scores on the VOSP silhouette, VOSP progressive silhouette, and WASI matrix reasoning tests in the at-risk group, but not controls.

### SD_BOLD_ correlates with demographic, brain structure, and cognitive variables

**Figure 3.**
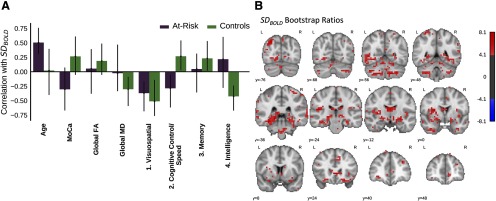
Multivariate PLS analysis of the relationship between SD_BOLD_ and 8 variables (age, MoCA score, global FA and MD, and score on 4 neuropsychological factors). In controls, SD_BOLD_ (particularly in the MTL) was associated with higher cognitive control/speed and intelligence scores, but lower visuospatial and memory scores. These relationships were weaker in the at-risk group. ***A***, The first latent variable (*p* < 0.001, 41.1% covariance) from the omnibus, between groups behavioral PLS assessing correlations between the 8 variables and SD_BOLD_. The bars represent the correlation between each variable with the pattern of SD_BOLD_ shown in the corresponding brain plot (***B***). The error bars represent 95% confidence intervals, so the error bars of variables significantly contributing to the latent variable will not cross zero. B Brain plots showing the bootstrap ratios for the latent variable, which may be interpreted like *z* scores. That is, the highlighted voxels are reliably associated with the related variables in *A* that significantly contribute to the latent variable. To clearly show the spatial pattern of the respective latent variable, only voxels with bootstrap ratios >|4| are pictured.

The omnibus behavioral PLS compared the relations between voxelwise SD_BOLD_ and several potentially related variables (age, MoCA score, global FA, global MD, and scores on our 4 cognitive factors) in both groups. A single significant latent variable was identified, *p *<* *0.001, 41.1% covariance. This LV (Fig. 3) showed that in controls, higher SD_BOLD_ was related to higher scores on the cognitive control/speed and intelligence factors, whereas lower SD_BOLD_ was related to greater global MD and higher scores on the visuospatial and memory factors. The voxels that most reliably related to this LV were located in the posterior medial temporal lobes, hippocampus, visual cortex, and striatum (Fig. 3B; Table 3). In the at-risk group, we observed the same pattern as in controls of lower SD_BOLD_ related to higher scores on the visuospatial factor. However, higher SD_BOLD_ was also related to older age and lower scores on the control/speed factor in the at-risk group. That is, the positive relation between the cognitive control/speed factor and SD_BOLD_ seen in the control group was reversed in the at-risk group. Notably, there was no relation between age and SD_BOLD_ in controls and no relation between memory and intelligence and SD_BOLD_ in the at-risk group.

**Table 3.** Significant clusters representing relationship between SD_BOLD_ and eight variables (Fig. 3)

ROI name	MNI coordinates, mm; *x*, *y*, *z*	Cluster size, voxels	BSR
Left parahippocampal gyrus	27, 22, 13	789	8.13
Right thalamus	17, 26, 16	369	7.31
Left lateral occipital cortex	31, 12, 26	32	7.02
Right caudate, subcallosal cortex	21, 36, 17	30	7.28
Left temporal fusiform cortex	31, 30, 8	29	5.72
Right superior parietal lobule	15, 21, 34	28	5.89
Right superior temporal gyrus, posterior division	6, 23, 18	28	7.09
Left superior parietal lobule	29, 19, 34	25	6.53
Left occipital pole	25, 8, 20	25	5.57
Left middle frontal gyrus	33, 36, 25	19	5.7
Left superior temporal gyrus	38, 23, 18	17	5.84
Left middle temporal gyrus	38, 28, 15	16	5.08
Right insular cortex	12, 29, 17	13	4.69
Right frontal pole	14, 42, 24	12	5.72
Left postcentral gyrus	33, 24, 30	11	5.25
Right precuneus cortex	21, 11, 28	11	5.65
Right paracingulate gyrus	20, 44, 16	10	5.56
Right superior paracingulate gyrus	20, 37, 27	10	4.59
Left caudate	25, 36, 20	8	5.23
Right frontal operculum cortex	13, 38, 18	8	4.95
Right angular gyrus	6, 19, 21	7	4.87
Right insular cortex	14, 32, 20	7	5.2
Right parietal operculum cortex	11, 25, 22	7	4.51
Left postcentral gyrus	38, 27, 24	7	5.42
Left juxstapositional lobule cortex (formerly supplementary morter cortex)	22, 28, 31	7	4.75
Left precuneus cortex	23, 18, 34	7	5.13
Left precuneus cortex	27, 16, 20	6	4.82
Right frontal pole	22, 46, 25	6	5.11
Right frontal pole	10, 33, 8	5	5.06
Right lateral occipital cortex, superior division	14, 15, 28	5	5.38
Right precuneus cortex	20, 18, 31	5	5.13

Only clusters of five voxels or larger with bootstrap ratio (BSR) >|4| are included.

### MTL SD_BOLD_ correlates with MTL volumes

**Figure 4.**
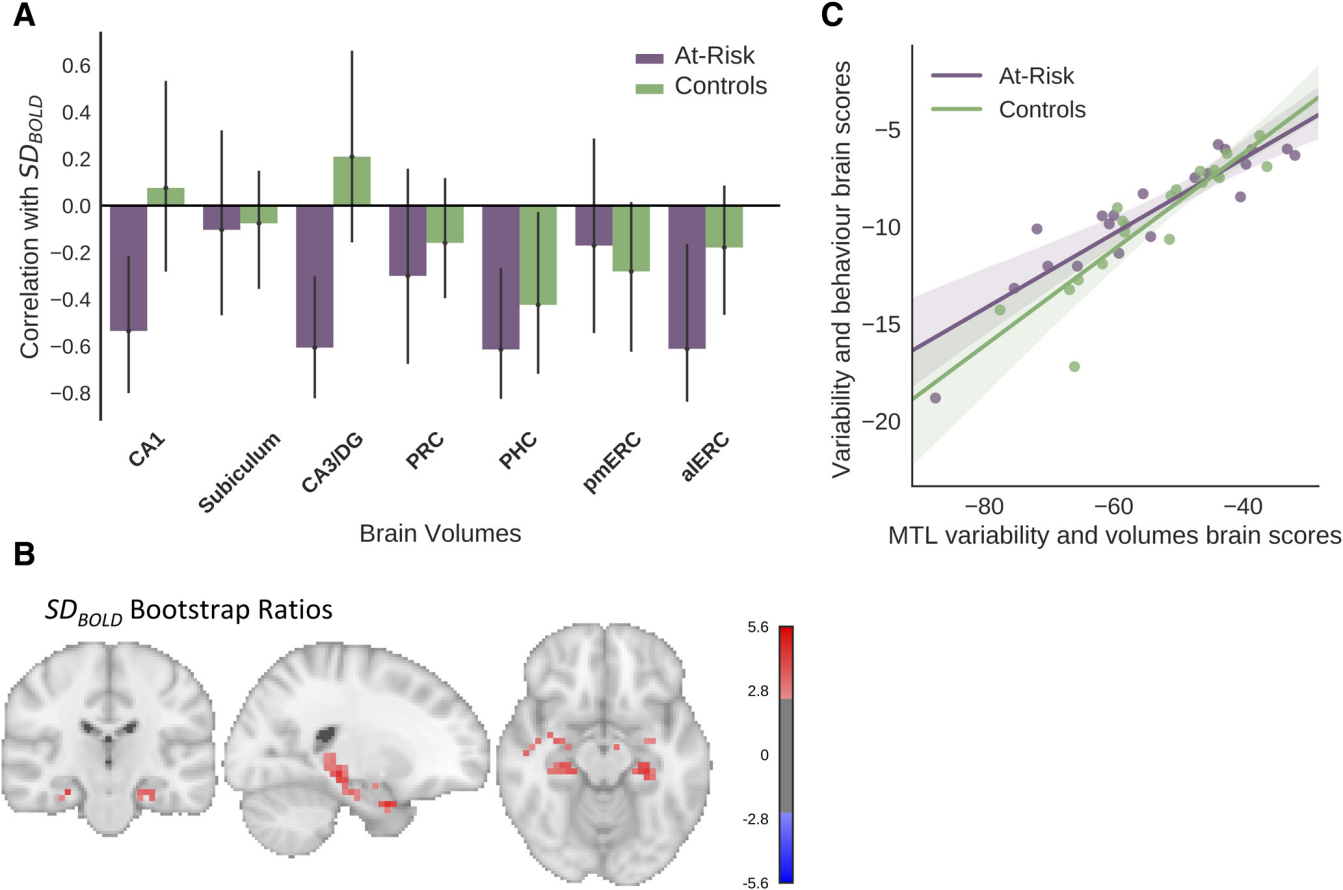
Multivariate PLS analysis of the relationship between SD_BOLD_ and gray matter volume in the HC and MTL. In the at-risk group, reduced volumetry in the CA1, CA3/DG, PHC, and alERC was associated with higher SD_BOLD_ in the MTL. The effect was less widespread in controls. ***A***, ***B***, The first latent variable (*p* < 0.0001, 72.0% covariance) from the omnibus between groups behavioral PLS assessing correlations between volumetry of 7 MTL regions (3 hippocampal subfields and 4 MTL subregions) and MTL SD_BOLD_. ***A***, The bars represent the correlation between each MTL subregion with the pattern of SD_BOLD_ shown in the corresponding brain plot (***B***). The error bars represent 95% confidence intervals, so the error bars of variables significantly contributing to the latent variable will not cross zero. ***B***, Brain plots showing the bootstrap ratios for the latent variable, which may be interpreted like *z* scores. Voxels with bootstrap ratios >|2.5| are pictured and considered to be reliably correlated with the brain volumes that are significantly contributing to the latent variable. ***C***, Scatter plot shows the correlation between brain scores from the PLS comparing whole-brain SD_BOLD_ and several predictor variables (Fig. 3) and the brain scores from the PLS comparing MTL variability and volumes. The strong correlation (at-risk: *r *=* *0.89, *p *<* *0.0001; controls: *r *=* *0.91, *p *<* *0.0001) indicates that the pattern of SD_BOLD_ that is associated with behavior is highly similar to the pattern of SD_BOLD_ associated with MTL volumes. Brain volumes: HC subfields (CA1, dentate gyrus/CA2 and 3, and subiculum) and MTL cortices (alERC, pmERC, PRC, and PHC).

An omnibus behavioral PLS compared bilateral MTL SD_BOLD_ with gray matter volume of three hippocampal subfields and four MTL cortices in both groups (Fig. 4*A*,*B*). A single significant LV (*p *<* *0.0001, 72.0% covariance) showed that reduced volume was associated with greater MTL SD_BOLD,_ especially in the at-risk group. Specifically, reduced volumes in the CA1, CA3/DG, PHC, and alERC were related to higher MTL SD_BOLD_ in the at-risk group. In the control group, the same pattern of greater MTL SD_BOLD_ was associated with lower PHC volume.

Brain scores from the PLS of MTL variability and volume were highly correlated with brain scores from the whole-brain variability and behavioral PLS in the control (r = 0.91, *p *<* *0.0001) and at-risk (*r* = 0.89, *p *<* *0.0001) groups (Fig. 4*C*). This suggests the pattern of SD_BOLD_ associated with behavioral performance is highly correlated with the pattern of SD_BOLD_ related to MTL volumes. Brain scores from the PLS comparing MTL variability and volumes (Fig. 4*A*,*B*) were not correlated with memory factor scores in both groups (at-risk: *r* = 0.13, *p* = 0.57; controls: *r* =−0.39, *p* = 0.09).

## Discussion

We investigated the relations between BOLD signal variability and cognitive performance in a group of healthy older adults and a group of older adults at-risk for cognitive decline, as documented by their sub-threshold MoCA score and reduced MTL volumes (Olsen et al., 2017). Our study demarcated older adults into at-risk and control groups based on MoCA score because the MoCA has demonstrated association with cognitive performance (D’Angelo et al., 2016; Yeung et al., 2017; Ryan et al., 2019), electrophysiological signature (Newsome et al., 2013), MTL volume (Olsen et al., 2017), strong sensitivity and specificity for MCI (Nasreddine et al., 2005; Markwick et al., 2012), and prediction of future conversion to AD (Julayanont et al., 2014). In healthy older adults greater SD_BOLD_ was associated with higher cognitive control/speed and intelligence scores, but lower memory and visuospatial scores. Notably, this effect was not as robust in the at-risk group. The at-risk group demonstrated neither the positive correlations observed between SD_BOLD_, cognitive control/speed and intelligence, nor the negative correlation between SD_BOLD_ and memory scores. The effect observed in controls was most robust in the MTL, where AD pathology is known to begin (Braak and Braak, 1991; Braak et al., 1993) and where our sample of at-risk individuals had significantly reduced volume (Olsen et al., 2017). We found that greater SD_BOLD_ in the MTL was correlated with lower parahippocampal volume in both groups, but that this relation involved more MTL regions in the at-risk group. Critically, we also found no group difference in SD_BOLD_ between groups. Together, our results suggest that cortical atrophy may lead to greater brain signal variability. Moreover, these changes may explain the differential relations observed between brain signal variability and cognitive control/speed performance in both groups. In the following we discuss implications for the role of SD_BOLD_ in cognition of healthy older adults and in those at-risk for developing MCI.

Our first analyses assessed correlations between SD_BOLD_ and several potentially related variables: age, MoCA score, global FA, global MD, and scores on our four cognitive factors. In controls, we found that greater BOLD signal variability, particularly in posterior MTL regions, was related to lower global MD, suggesting generally greater white matter integrity. In terms of cognition, greater BOLD signal variability was related to better performance on the cognitive control/speed and intelligence factors, but poorer performance on the visuospatial and memory factors. Our results corroborated previous work, which found that the direction of the relationship between SD_BOLD_ and cognitive factors was dependent on cognitive domain and correlated with global white matter integrity in healthy older adults (Burzynska et al., 2015). The cognitive control/speed and intelligence factors may have required greater dynamic range than visuospatial and memory factors, driving the different relations with SD_BOLD_, as first hypothesized by Burzynska et al., 2015.

The regions where we observed a correlation between SD_BOLD_ and cognition have been associated with various cognitive functions. Briefly, the bilateral superior parietal regions are primarily associated with voluntary control of attention during perception, but may also be involved with memory retrieval, monitoring, and verification (Cabeza et al., 2008). The hippocampus is broadly understood to support episodic memory (Moscovitch et al., 2016). The thalamus relays sensory and motor signals to the cerebral cortex and is implicated in consciousness and sleep (Steriade and Llinás, 1988). The fusiform and inferior temporal cortex are involved in the ventral visual stream and shape processing (Haxby et al., 1991; Denys et al., 2004). Last, the insular cortex has multiple functions including multimodal sensory processing and binding (Bushara et al., 2003) and a role in emotion (Phan et al., 2002). A similarity among many of these regions is that they have been identified as network hubs (van den Heuvel and Sporns, 2011). Hubs are identified as well-connected regions through structural or functional network analyses. As was initially proposed by Burzynska et al. (2015), we suggest that the high connectivity of these regions may result in or require greater neural variability, which would be reflected by greater SD_BOLD_. That is, greater SD_BOLD_ in these regions might indicate more flexible network integration, which presumably would support higher scores on the cognitive control/speed and intelligence factors.

We report a negative association between MTL SD_BOLD_ and our memory factor, whereas Burzynska et al. (2015) reported a positive relationship and other studies have shown SD_BOLD_ supports other types of cognitive function (Garrett et al., 2011, 2013a). However, another recent study found a negative association between SD_BOLD_ and memory scores, though this was in a sample of AD patients (Scarapicchia et al., 2018). Regardless, the discrepancy highlights the need for further study with larger samples and standardized methodology. We also note the possibility that differences in the neuropsychological battery and preprocessing choices may contribute to the observed inconsistencies across the literature. The memory factors of the present study, Scarapicchia et al. (2018) and Burzynska et al. (2015) were composed of different tests, which require use of different cognitive constructs to varying degrees (e.g., in the use of targets that tax verbal vs visuospatial ability). Additionally, we note that the present study and Burzynska et al. (2015) de-noised the fMRI data, but Scarapicchia et al. (2018) did not, and prior work has shown the importance of removing as much unwanted noise as possible when examining SD_BOLD_ (Garrett et al., 2010; Grady and Garrett, 2014).

Our initial omnibus PLS found that older age, and lower scores on the visuospatial and cognitive control/speed factors were related to greater BOLD signal variability in the at-risk group. The positive relationship between visuospatial performance and SD_BOLD_ was common in the at-risk and control groups. In the at-risk group however, SD_BOLD_ also correlated positively with age. Earlier work observed that SD_BOLD_ in fMRI task-based contexts was reduced in older age (Garrett et al., 2011, 2013b; Grady and Garrett, 2014, 2018). However, as we observed here, some of these studies found isolated brain regions with increased SD_BOLD_ during rest and task-fMRI associated with age (Garrett et al., 2010, 2011; Nomi et al., 2017). Most notably, Garrett et al. (2010) found age-related increases in the MTL, paralleling our finding in the at-risk group. Therefore, although this relation between SD_BOLD_ and age was somewhat weak in the at-risk group, the effect was nevertheless consistent with prior work. The group difference in age-SD_BOLD_ correlation was unexpected, but likely reflects greater age-related variation in the neuropsychological battery (Fig. 2) in the at-risk group, rather than difference in SD_BOLD_. We advocate multivariate techniques such as PLS and canonical correlation, which can unmask rich relations between age, other demographic variables, and brain function (Ferreira et al., 2016; Perry et al., 2017).

Critically, we found the at-risk group did not show the positive correlations observed in controls between intelligence, cognitive control/speed, and SD_BOLD_. Whereas controls showed a positive relationship between control/speed and SD_BOLD_, the at-risk group showed a negative relationship. Similarly, controls showed a positive relationship between intelligence and SD_BOLD_, but this relation was non-significant in the at-risk group. Additionally, we found a negative relationship in controls between memory performance and SD_BOLD_, whereas this effect was absent in the at-risk group. Our results suggest that alterations in the relations between cognitive performance and SD_BOLD_ may occur in individuals at-risk for further cognitive decline, including MCI. Importantly, the effect was most robust in the MTL, where AD pathology is known to begin (Braak and Braak, 1991; Braak et al., 1993), and where this sample of ostensibly healthy older adults showed reduction in gray matter volume (Olsen et al., 2017).

In both groups, lower parahippocampal volume was associated with greater SD_BOLD_ in MTL brain areas. Interestingly, the effect was more widespread in the at-risk group, where lower volume of the CA1, CA3/DG, and alERC were also related to greater SD_BOLD_. Importantly, a previous study with our sample found that reduced volume in the MTL, especially the alERC, CA1/DG, and PRC were strongly associated with MoCA performance (Olsen et al., 2017). Furthermore, volume in the alERC has been linked to object processing in this sample (Yeung et al., 2017). Together, these results suggest lower MTL volume is likely related to greater SD_BOLD_ in the MTLs of older adults regardless of cognitive status; however, the relationship was more reliable in the at-risk group, possibly because of greater variability in MTL subregion volumes. Furthermore, the correlation between brain scores from both PLS analyses (Figs. 3. 4), suggests a strong similarity between the patterns of SD_BOLD_ associated with MTL volumes and behavioral performance. In fact, we found a trend for a similar negative correlation between the brain scores from the MTL variability and volumes PLS and the memory factor in controls, but not the at-risk group, similar to what was observed in the SD_BOLD_ and behavior PLS (Fig. 4). Given the exclusively negative associations between SD_BOLD_ and cognitive performance in the at-risk group, greater variability may represent a failed attempt to compensate for cortical atrophy in the MTL. In contrast, the controls showed positive associations between SD_BOLD_ and the cognitive control/speed and intelligence factors, possibly because they have better maintained MTL volumes.

Despite finding robust group differences in the relations between SD_BOLD_ and our demographic and behavioral variables across groups, we did not observe a group difference in SD_BOLD_. Patients with various clinical diagnoses including stroke (Kielar et al., 2016), multiple sclerosis (Petracca et al., 2017), 22q11.2 deletion syndrome (Zöller et al., 2017), and AD (Scarapicchia et al., 2018) have demonstrated greater SD_BOLD_ relative to comparison groups. However, our sample of at-risk participants, who presented with only early warning signs of cognitive decline that did not meet diagnostic criteria for MCI, did not show a difference in SD_BOLD_. We acknowledge SD_BOLD_ is sensitive to decisions made when preprocessing the raw data (Garrett et al., 2010; Turner et al., 2015). To this end, we highlight the careful de-noising of our data, which we view as an important step for analyses of SD_BOLD_, as it has been shown to enhance age effects (Garrett et al., 2010) and should function to reduce the influence of “junk” noise. If de-noising enhances group differences then we can have confidence in our finding of no group differences in SD_BOLD_ per se, as de-noising should have made it easier to such differences if they existed.

We acknowledge the cognitive factors used this study require contribution from multiple cognitive domains. As such, future studies should aim to pinpoint the relations between specific cognitive functions and SD_BOLD_ and how these associations may change in disease. We also recognize our small sample size (*N* = 40) limits the generalizability of our findings, and highlight the need for longitudinal studies that follow the “at-risk” group. Last, variability of resting-state SD_BOLD_ is contentious, particularly in the context of aging (Tsvetanov et al., 2015, 2019). In this regard, we note that our study did not focus exclusively on age effects and again highlight our careful de-noising of the data, which addresses artifact from cardiovascular and neurovascular signal.

### Conclusions

We found that a group of ostensibly healthy older adults previously identified as at-risk (Olsen et al., 2017) showed a different relation between SD_BOLD_ and cognition than did controls. In controls, SD_BOLD_ was positively correlated with tasks of cognitive control/speed and intelligence, but negatively correlated with memory and visuospatial scores. In contrast, these relations were weaker in the at-risk group. Notably, the relations between SD_BOLD_ and cognition observed in controls were most robust in MTL regions, where AD pathology first occurs (Braak and Braak, 1991; Braak et al., 1993). In addition, we showed that both groups had a negative relation between parahippocampal volume and MTL SD_BOLD_, however, the effect was more widespread in the at-risk group. Our findings provide evidence that brain signal variability may increase in the face of cortical atrophy, leading to the differential relations observed between brain signal variability and cognitive performance in groups of different cognitive status.

10.1523/ENEURO.0290-19.2020.t2-2 Table 2-2 Supplementary Table 2-2. Download Table 2-2, DOCX file.

## Synthesis

Reviewing Editor: Karen Davis, Krembil Research Institute and University of Toronto Decisions are customarily a result of the Reviewing Editor and the peer reviewers coming together and discussing their recommendations until a consensus is reached. When revisions are invited, a fact-based synthesis statement explaining their decision and outlining what is needed to prepare a revision will be listed below. The following reviewer(s) agreed to reveal their identity: Jason Nomi.

The authors investigate brain signal variability changes and its association with multiple phenotypic and imaging measures in older adults with and without risk for cognitive decline. Specifically, the study examined the relationship between SDbold variability, mild cognitive impairment, cognitive tests, and structural diffusion measures.

The results show that SDbold in the MTL is related to cognitive performance and MTL volume in both groups. Additionally, SDbold in the MTL and occipital lobe were positively related to cognitive control tasks but negatively related to memory performance in both groups with this association being weaker in the mild cognitive impairment group. Specifically, multivariate analysis reveals a surprising dissociable relationship between brain signal variability and cognitive domains across individuals - greater signal variability shows an expected positive association with increased intelligence and “cognitive control” performance, whilst decreased variability predominately in temporal areas is interestingly related to greater memory scores. Subtle differences in the brain-behavior relations between those with and without risk for cognitive decline, are suggested by the authors as evidence for “pathological changes” in the at-risk group which “disrupt” normal age-related cognitive changes.

The study is well written, well conducted, and could be of interest to clinicians interested in fMRI variability research. The strengths include the use of multi-modal imaging measures and multivariate analyses. However, the small-sample, cross-sectional-design and also methodological issues throughout the study dampen enthusiasm for the study and limit the interpretation of these findings.

Other Issues and Comments:

1. Were there any group differences in head motion? These stats should be included. Correlations between MTL volume and head motion should also be noted.

2. Why was randomize used for the group difference in FA/MD? From what I understand, couldn’t these also be subjected to a PLS analysis? Are there any differences in voxel-wise FA/MD using a PLS approach? It seems like the same statistical test should be used to investigate group differences so the assumptions are the same.

3. The authors should address tThe demarcation of older individuals into those with and without risk for cognitive decline because these groups do not show significant differences across the cognitive factors. Could the authors a) elaborate on why the MoCA is potentially more sensitive to determine an individual’s “risk”? b) provide group differences in the neuropsychological battery measures,.c) illustrate the scatter distributions for the MoCA score across groups? d) note whether the groups show differences on the individual test scores, and/or whether these tests reveal differential cross-sectional changes with age?

4. The manuscript would benefit from reporting the basic demographic and phenotypic characteristics (and ideally here within a table) of the two groups.

5. There were no clear group differences in the large-scale neural and cognitive data (apart from the MoCA scores) and given the cross-sectional approach, I revising interpreting the findings in the at-risk group as reflecting “pathological changes..” and also brain-behavior relations that are “disrupted” is unsubstantiated. Differential associations could be related to differences in variance of BOLD and cognitive data within the two-groups. Furthermore, the cross-sectional nature of the design precludes the currently used terminology of “volume loss” and this needs to be addressed. Lastly, what are the test-statistics for the MTL volumetric differences between the two groups that was previously performed, and have any basic group-comparisons for the sub-regions been performed here?

6. A few questions arise with the grouping of neuropsychological tests and their cognitive labels. Firstly, it’s worth noting that the four tests denoted as “cognitive control” assess multi-factorial cognitive components. For instance, the TMT A and Digit Span forward tests are commonly recognized as tapping into lower-level attention and processing speed abilities (Rosenthal et al., 2006; Tsang et al., 2013). Whereas the DigitSpan backwards and TMT B account primarily for variance in executive functioning. Secondly, many of the other neuropsychological tests in the current study are also multi-factorial in the cognitive processes they express. The contribution of each individual test to the latent PLS factor - relative to the other tests which also load strongly upon the cognitive component - should be ascertained. This can be done through the PLS output correlating the individual test scores with each individual’s brain scores (i.e. variable U in the PLS output) (Zeighami et al., 2019).

7. The study is underpowered which has implications for the large number of variables included in the PLS and also the within-group analyses. I recommend that the authors address the “behavioral” variables selected (in both the main and post-hoc analyses) as this will evidently influence the linear weighting of variables that maximally co-vary with each other. Given the two elderly groups do not demonstrate significant white-matter differences, it’s first unclear why the FA and MD measures were included in the main PLS model. Moreover, there are almost half the number of “behavioral variables” in comparison to the number of individuals for the within-group analyses. Generally, it is recommended that the number of observations in multivariate models are roughly ten-times in magnitude to the number of variables. I would suggest the within-group analyses are removed, unless the authors can address the optimal number of variables to include. Consequently, the authors could address their interpretations that decreased MTL volume is related to increased variability in both cognitive groups.

8. The post-hoc analysis of latent structure-function relationships in the medial temporal lobe should also determine the association with memory performance. This does not mean including additional memory performance scores in the PLS model, but correlating the component (or test) scores with mean brain scores - for an application see Fig 5 from (Burzynska et al., 2013).

9. There is a focus on the functional variability of the temporal lobe and associations with memory performance. However, the brain regions contributing reliably to the latent brain-behavior relationships are distributed across the brain. As a minimum, the authors should report in a table these regions along with their basic spatial information (i.e. structure name, MNI coordinates) and also improve the brain heat map visualization of the BSR magnitudes. The discussion should contain additional interpretations of these non-medial temporal lobe areas (being cautious about issues with reverse inference), and their relevance to the behavioral factors expressed by the latent variable (i.e. cognitive control and intelligence). Lastly, what is the relevance of the specific medial temporal subfields that demonstrate associations between volume size and behavior?

10. There could be more interpretation of the associations of variability with behavioral performance, particularly in reference to non-memory domains and the direction of the findings. Can the authors offer any interpretation for these discrepancies, such as differences in the age-groups, neuropsychological batteries, or other methodologies? Moreover, there should be further interpretation of the striking differential brain-behavior relationships between the two groups for age and cognitive control factors.

11. A few other misleading statements have been made in the discussion and should be revised. First, given that increased functional integration in older age is a surprising yet robust finding across the literature (Damoiseaux, 2017), increased resting-state variability may perhaps be related to sub-optimal patterns of functional integration. However, there’s no evidence from the current findings to suggest differential relationships between variability and integration across the two groups (Line 428), nor at least basic differences in variability. Next, any reference to mechanisms and pathological changes should be addressed, as this terminology in too strong in light of the current findings and cross-sectional design. Lastly, I’m not sure of the authors intended message in regards to the importance of appropriately denoising their data? - Are they trying to suggest no group differences are appropriately found given they have rigorously denoised their data?

12. Were any individuals removed from the study/analysis due to poor quality imaging data? Another important consideration is head-motion.

13. For any reference to the literature upon BOLD variability and ageing, whether the fMRI data were collected during a task-free or task-design needs to be indicated.

14. Additional citations include: multivariate analyses in the context of ageing (Ferreira et al., 2016; Perry et al., 2017) and this review paper (Damoiseaux, 2017) , especially in context of the functional integration changes with age.

References

1. Burzynska, A.Z., Garrett, D.D., Preuschhof, C., Nagel, I.E., Li, S.-C., Bäckman, L., Heekeren, H.R., Lindenberger, U., 2013. A scaffold for efficiency in the human brain. Journal of neuroscience 33, 17150-17159.

2. Damoiseaux, J.S., 2017. Effects of aging on functional and structural brain connectivity. Neuroimage 160, 32-40.

3. Ferreira, L.K., Regina, A.C.B., Kovacevic, N., Martin, M.d.G.M., Santos, P.P., Carneiro, C.d.G., Kerr, D.S., Amaro Jr, E., McIntosh, A.R., Busatto, G.F., 2016. Aging effects on whole-brain functional connectivity in adults free of cognitive and psychiatric disorders. Cerebral cortex 26, 3851-3865.

4. Perry, A., Wen, W., Kochan, N.A., Thalamuthu, A., Sachdev, P.S., Breakspear, M., 2017. The independent influences of age and education on functional brain networks and cognition in healthy older adults. Human brain mapping 38, 5094-5114.

5. Rosenthal, E.N., Riccio, C.A., Gsanger, K.M., Jarratt, K.P., 2006. Digit Span components as predictors of attention problems and executive functioning in children. Archives of Clinical Neuropsychology 21, 131-139.

6. Tsang, R.S., Sachdev, P.S., Reppermund, S., Kochan, N.A., Kang, K., Crawford, J., Wen, W., Draper, B., Trollor, J.N., Slavin, M.J., 2013. Sydney Memory and Ageing Study: an epidemiological cohort study of brain ageing and dementia. International review of psychiatry 25, 711-725.

7. Zeighami, Y., Fereshtehnejad, S.M., Dadar, M., Collins, D.L., Postuma, R.B., Misic, B., Dagher, A., 2019. A clinical-anatomical signature of Parkinson’s disease identified with partial least squares and magnetic resonance imaging. Neuroimage 190, 69-78.

## References

[B1] Alzheimer’s Association (2017) 2017 Alzheimer’s disease facts and figures. Alzheimers Dement 13:325–373.

[B2] Beckmann CF, Smith SM (2004) Probabilistic independent component analysis for functional magnetic resonance imaging. IEEE Trans Med Imaging 23:137–152. 10.1109/TMI.2003.822821 14964560

[B3] Braak H, Braak E (1991) Neuropathological stageing of Alzheimer-related changes. Acta Neuropathol 82:239–259. 10.1007/bf00308809 1759558

[B4] Braak H, Braak E, Bohl J (1993) Staging of Alzheimer related cortical destruction. Eur Neurol 33:403–408. 10.1159/000116984 8307060

[B5] Burzynska AZ, Wong CN, Voss MW, Cooke GE, McAuley E, Kramer AF (2015) White matter integrity supports BOLD signal variability and cognitive performance in the aging human brain. PLoS One 10:e0120315–17. 10.1371/journal.pone.0120315 25853882 PMC4390282

[B6] Bushara KO, Hanakawa T, Immisch I, Toma K, Kansaku K, Hallett M (2003) Neural correlates of cross-modal binding. Nat Neurosci 6:190–195. 10.1038/nn993 12496761

[B7] Cabeza R, Ciaramelli E, Olson IR, Moscovitch M (2008) Parietal cortex and episodic memory: an attentional account. Nat Rev Neurosci 9:613–625. 10.1038/nrn2459 18641668 PMC2692883

[B8] Damoiseaux JS (2017) Effects of aging on functional and structural brain connectivity. Neuroimage 160:32–40. 10.1016/j.neuroimage.2017.01.077 28159687

[B9] D’Angelo MC, Smith VM, Kacollja A, Zhang F, Binns MA, Barense MD, Ryan JD (2016) The effectiveness of unitization in mitigating age-related relational learning impairments depends on existing cognitive status. Neuropsychol Dev Cogn B Aging Neuropsychol Cogn 23:667–690. 10.1080/13825585.2016.1158235 27049878 PMC4926786

[B10] Denys K, Vanduffel W, Fize D, Nelissen K, Peuskens H, Van Essen D, Orban GA (2004) The processing of visual shape in the cerebral cortex of human and nonhuman primates: a functional magnetic resonance imaging study. J Neurosci 24:2551–2565. 10.1523/JNEUROSCI.3569-03.2004 15014131 PMC6729498

[B11] Dubois B, Hampel H, Feldman HH, Scheltens P, Aisen P, Andrieu S, Bakardjian H, Benali H, Bertram L, Blennow K, Broich K, Cavedo E, Crutch S, Dartigues JF, Duyckaerts C, Epelbaum S, Frisoni GB, Gauthier S, Genthon R, Gouw AA (2016) Preclinical Alzheimer’s disease: definition, natural history, and diagnostic criteria. Alzheimers Dement 12:292–323. 27012484 10.1016/j.jalz.2016.02.002 PMC6417794

[B12] Ferreira LK, Regina ACB, Kovacevic N, da Graça Morais Martin M, Santos PP, Carneiro C d G, Kerr DS, Amaro E, McIntosh AR, Busatto GF (2016) Aging effects on whole-brain functional connectivity in adults free of cognitive and psychiatric disorders. Cereb Cortex 26:3851–3865. 10.1093/cercor/bhv190 26315689

[B13] Garrett DD, Kovacevic N, McIntosh AR, Grady CL (2010) Blood oxygen level-dependent signal variability is more than just noise. J Neurosci 30:4914–4921. 10.1523/JNEUROSCI.5166-09.2010 20371811 PMC6632804

[B14] Garrett DD, Kovacevic N, McIntosh AR, Grady CL (2011) The importance of being variable. J Neurosci 31:4496–4503. 10.1523/JNEUROSCI.5641-10.2011 21430150 PMC3104038

[B15] Garrett DD, Kovacevic N, McIntosh AR, Grady CL (2013a) The modulation of BOLD variability between cognitive states varies by age and processing speed. Cereb Cortex 23:684–693. 10.1093/cercor/bhs055 22419679 PMC3823571

[B16] Garrett DD, Samanez-Larkin GR, MacDonald SWS, Lindenberger U, McIntosh AR, Grady CL (2013b) Moment-to-moment brain signal variability: a next frontier in human brain mapping? Neurosci Biobehav Rev 37:610–624. 10.1016/j.neubiorev.2013.02.015 23458776 PMC3732213

[B17] Garrett DD, Nagel IE, Preuschhof C, Burzynska AZ, Marchner J, Wiegert S, Jungehülsing GJ, Nyberg L, Villringer A, Li SC, Heekeren HR, Bäckman L, Lindenberger U (2015) Amphetamine modulates brain signal variability and working memory in younger and older adults. Proc Natl Acad Sci U S A 112:7593–7598. 10.1073/pnas.1504090112 26034283 PMC4475975

[B18] Garrett DD, Epp SM, Perry A, Lindenberger U (2018) Local temporal variability reflects functional integration in the human brain. Neuroimage 183:776–787. 10.1016/j.neuroimage.2018.08.019 30149140

[B19] Grady CL, Garrett DD (2014) Understanding variability in the BOLD signal and why it matters for aging. Brain Imaging Behav 8:274–283. 10.1007/s11682-013-9253-0 24008589 PMC3922711

[B20] Grady CL, Garrett DD (2018) Neuroimage Brain signal variability is modulated as a function of internal and external demand in younger and older adults. Neuroimage 169:510–523. 10.1016/j.neuroimage.2017.12.031 29253658

[B21] Guitart-masip M, Salami A, Garrett D, Rieckmann A, Lindenberger U, Bäckman L (2016) BOLD Variability is related to dopaminergic neurotransmission and cognitive aging. Cereb Cortex 26:2074–2083. 10.1093/cercor/bhv029 25750252

[B22] Haxby JV, Grady CL, Horwitz B, Ungerleider LG, Mishkin M, Carson RE, Herscovitch P, Schapiro MB, Rapoport SI (1991) Dissociation of object and spatial visual processing pathways in human extrastriate cortex. Proc Natl Acad Sci U S A 88:1621–1625. 10.1073/pnas.88.5.1621 2000370 PMC51076

[B23] Jack Jr CR, Holtzman DM (2013) Biomarker modeling of Alzheimer’s disease. Neuron 80:1347–1358. 24360540 10.1016/j.neuron.2013.12.003 PMC3928967

[B24] Jack Jr CR, Knopman DS, Jagust WJ, Petersen RC, Weiner MW, Aisen PS, Shaw LM, Vemuri P, Wiste HJ, Weigand SD, Lesnick TG, Pankratz VS, Donohue MC, Trojanowski JQ (2013) Tracking pathophysiological processes in Alzheimer’s disease: an updated hypothetical model of dynamic biomarkers. The Lancet Neurology 12:207–216. 23332364 10.1016/S1474-4422(12)70291-0 PMC3622225

[B25] Jenkinson M, Bannister P, Brady M, Smith S (2002) Improved optimization for the robust and accurate linear registration and motion correction of brain images. Neuroimage 17:825–841. 10.1016/S1053-8119(02)91132-8 12377157

[B26] Julayanont P, Brousseau M, Chertkow H, Phillips N, Nasreddine ZS (2014) Montreal Cognitive Assessment Memory Index Score (MoCA-MIS) as a predictor of conversion from mild cognitive impairment to Alzheimer’s disease. J Am Geriatr Soc 62:679–684. 10.1111/jgs.12742 24635004

[B27] Kielar A, Deschamps T, Chu RKO, Jokel R, Khatamian YB, Chen JJ, Meltzer JA (2016) Identifying dysfunctional cortex: dissociable effects of stroke and aging on resting state dynamics in MEG and fmri. Front Aging Neurosci 8:40. 10.3389/fnagi.2016.00040 26973515 PMC4776400

[B28] Markwick A, Zamboni G, De Jager CA (2012) Profiles of cognitive subtest impairment in the Montreal Cognitive Assessment (MoCA) in a research cohort with normal Mini-Mental State Examination (MMSE) scores. J Clin Exp Neuropsychol 34:750–757. 10.1080/13803395.2012.672966 22468719

[B29] McIntosh AR, Lobaugh NJ (2004) Partial least squares analysis of neuroimaging data: applications and advances. Neuroimage 23:S250–S263. 10.1016/j.neuroimage.2004.07.020 15501095

[B30] McIntosh AR, Mišić B (2013) Multivariate statistical analyses for neuroimaging data. Annu Rev Psychol 64:499–525. 10.1146/annurev-psych-113011-143804 22804773

[B31] Moscovitch M, Cabeza R, Winocur G, Nadel L (2016) Episodic memory and beyond: the hippocampus and neocortex in transformation. Annu Rev Psychol 67:105–134. 10.1146/annurev-psych-113011-143733 26726963 PMC5060006

[B32] Nasreddine ZS, Phillips NA, Bedirian V, Charbonneau S, Whitehead V, Collin I, Cummings JL, Chertkow H (2005) The Montreal Cognitive Assessment, MoCA: a brief screening tool for mild cognitive impairment. J Am Geriatr Soc 53:695e699. 15817019 10.1111/j.1532-5415.2005.53221.x

[B33] Newsome RN, Pun C, Smith VM, Ferber S, Barense MD (2013) Neural correlates of cognitive decline in older adults at-risk for developing MCI: evidence from the CDA and P300. Cogn Neurosci 4:152–162. 10.1080/17588928.2013.853658 24251603

[B34] Nomi JS, Bolt TS, Ezie C, Uddin LQ, Heller AS (2017) Moment-to-moment BOLD signal variability reflects regional changes in neural flexibility across the lifespan. J Neurosci 37:5539–5548. 10.1523/JNEUROSCI.3408-16.2017 28473644 PMC5452342

[B35] Olsen RK, Nichols EA, Chen J, Hunt JF, Glover GH, Gabrieli JDE, Wagner AD (2009) Performance-related sustained and anticipatory activity in human medial temporal lobe during delayed match-to-sample. J Neurosci 29:11880–11890. 10.1523/JNEUROSCI.2245-09.2009 19776274 PMC2775810

[B36] Olsen RK, Palombo DJ, Rabin JS, Levine B, Ryan JD, Rosenbaum RS (2013) Volumetric analysis of medial temporal lobe subregions in developmental amnesia using high-resolution magnetic resonance imaging. Hippocampus 23:855–860. 10.1002/hipo.22153 23749334 PMC4165307

[B37] Olsen RK, Yeung LK, Noly-Gandon A, D’Angelo MC, Kacollja A, Smith VM, Ryan JD, Barense MD (2017) Human anterolateral entorhinal cortex volumes are associated with cognitive decline in aging prior to clinical diagnosis. Neurobiol Aging 57:195–205. 10.1016/j.neurobiolaging.2017.04.025 28578804

[B38] Osterreith PA (1944) Le test de copie d’une figure complex: contribution à l’étude de la perception et de la memoir. Arch Psychol 30:206–356.

[B39] Palombo DJ, Amaral RSC, Olsen RK, Muller DJ, Todd RM, Anderson AK, Levine B (2013) KIBRA polymorphism is associated with individual differences in hippocampal subregions: evidence from anatomical segmentation using high-resolution MRI. J Neurosci 33:13088–13093. 10.1523/JNEUROSCI.1406-13.2013 23926262 PMC6619733

[B40] Perry A, Wen W, Kochan NA, Thalamuthu A, Sachdev PS, Breakspear M (2017) The independent influences of age and education on functional brain networks and cognition in healthy older adults. Hum Brain Mapp 38:5094–5114. 10.1002/hbm.23717 28685910 PMC6866868

[B41] Petracca M, Saiote C, Bender HA, Arias F, Farrell C, Magioncalda P, Martino M, Miller A, Northoff G, Lublin F, Inglese M (2017) Synchronization and variability imbalance underlie cognitive impairment in primary-progressive multiple sclerosis. Sci Rep 7:46411–46412. 10.1038/srep46411 28429774 PMC5399449

[B42] Phan KL, Wager T, Taylor SF, Liberzon I (2002) Functional neuroanatomy of emotion: a meta-analysis of emotion activation studies in PET and fMRI. Neuroimage 16:331–348. 10.1006/nimg.2002.1087 12030820

[B43] Protzner AB, Kovacevic N, Cohn M, Mcandrews MP (2013) Characterizing functional integrity: intraindividual brain signal variability predicts memory performance in patients with medial temporal lobe epilepsy. J Neurosci 33:9855–9865. 10.1523/JNEUROSCI.3009-12.2013 23739982 PMC6619707

[B44] Reitan RM, Wolfson D (1985) The Halstead–Reitan neuropsychological test battery: theory and clinical interpretation, Vol. 4 Tucson, AZ; Neuropsychology.

[B45] Ryan JD, Kacollja A, D’Angelo MC, Newsome RN, Gardner S, Rosenbaum RS (2019) Existing semantic knowledge provides a schematic scaffold for inference in early cognitive decline, but not in amnestic MCI. Cognitive Neuropsychology. Cogn Neuropsychol. doi: 10.1080/02643294.2019. 10.1080/02643294.2019.1684886 31722612

[B46] Scarapicchia V, Mazerolle EL, Fisk JD, Ritchie LJ, Gawryluk JR (2018) Resting state BOLD variability in Alzheimer’s disease: a marker of cognitive decline or cerebrovascular status? Front Aging Neurosci 10:39 10.3389/fnagi.2018.00039 29515434 PMC5826397

[B47] Smith SM (2002) Fast robust automated brain extraction. Hum Brain Mapp 17:143–155. 10.1002/hbm.10062 12391568 PMC6871816

[B48] Smith SM, Jenkinson M, Johansen-Berg H, Rueckert D, Nichols TE, Mackay CE, Watkins KE, Ciccarelli O, Cader MZ, Matthews PM, Behrens TEJ (2006) Tract-based spatial statistics: voxelwise analysis of multi-subject diffusion data. Neuroimage 31:1487–1505. 10.1016/j.neuroimage.2006.02.024 16624579

[B49] Steriade M, Llinás RR (1988) The functional states of the thalamus and the associated neuronal interplay. Physiol Rev 68:649–742. 10.1152/physrev.1988.68.3.649 2839857

[B50] Tabachnick BG, Fidell LS, (2007) Using multivariate statistics 5th ed. Allyn & Bacon, Boston, MA.

[B51] Tsvetanov KA, Henson RNA, Tyler LK, Davis SW, Shafto MA, Taylor JR, Rowe JB (2015) The effect of ageing on fMRI: correction for the confounding effects of vascular reactivity evaluated by joint fMRI and MEG in 335 adults. Hum Brain Mapp 36:2248–2269. 10.1002/hbm.22768 25727740 PMC4730557

[B52] Tsvetanov KA, Henson RN, Jones PS, Mutsaerts HJ, Fuhrmann D, Tyler LK, Rowe JB; Cam-CAN (2019) The effects of age on resting-state BOLD signal variability is explained by cardiovascular and neurovascular factors. BioRxiv. https://doi.org/10.1101/836619. 10.1101/836619 PMC8244027 33210312

[B53] Turner BO, Lopez B, Santander T, Miller MB (2015) One dataset, many conclusions: BOLD variability’s complicated relationships with age and motion artifacts. Brain Imaging Behav 9:115–127. 10.1007/s11682-014-9351-7 25573194

[B54] Tzourio-Mazoyer N, Landeau B, Papathanassiou D, Crivello F, Etard O, Delcroix N, Mazoyer B, Joliot M (2002) Automated anatomical labeling of activations in SPM using a macroscopic anatomical parcellation of the MNI MRI single-subject brain. Neuroimage 15:273–289. 10.1006/nimg.2001.0978 11771995

[B55] van den Heuvel MP, Sporns O (2011) Rich-club organization of the human connectome. J Neurosci 31:15775–15786. 10.1523/JNEUROSCI.3539-11.2011 22049421 PMC6623027

[B56] Warrington EK, James M (1991) A new test of object decision: 2D silhouettes featuring a minimal view. Cortex 27:377–383. 10.1016/S0010-9452(13)80033-0 1743033

[B57] Wechsler D (1999) Manual for the Wechsler abbreviated intelligence scale (WASI). San Antonio: Psychological.

[B58] Wechsler D (2009) Wechsler memory scale (WMS-iv). New York: Psychological.

[B59] Wechsler D, Coalson D, Raiford S (2008) WAIS-iv: Wechsler adult intelligence scale. San Antonio: Pearson.

[B60] Yeung LK, Olsen RK, Bild-Enkin HEP, D’Angelo MC, Kacollja A, McQuiggan DA, Keshabyan A, Ryan JD, Barense MD (2017) Anterolateral entorhinal cortex volume predicted by altered intra-item configural processing. J Neurosci 37:5527–5538. 10.1523/JNEUROSCI.3664-16.2017 28473640 PMC6596534

[B61] Yushkevich PA, Amaral RS, Augustinack JC, Bender AR, Bernstein JD, Boccardi M, Bocchetta M, Burggren AC, Carr VA, Chakravarty MM, Chételat G, Daugherty AM, Davachi L, Ding SL, Ekstrom A, Geerlings MI, Hassan A, Huang Y, Iglesias JE, La Joie R, et al. (2015) Quantitative comparison of 21 protocols for labeling hippocampal subfields and parahippocampal subregions in *in vivo* MRI: towards a harmonized segmentation protocol. Neuroimage 111:526–541. 10.1016/j.neuroimage.2015.01.004 25596463 PMC4387011

[B62] Zöller D, Schaer M, Scariati E, Padula MC, Eliez S, Van De Ville D (2017) Disentangling resting-state BOLD variability and PCC functional connectivity in 22q11.2 deletion syndrome. Neuroimage 149:85–97. 10.1016/j.neuroimage.2017.01.064 28143774

