# Erratum: Good et al., “Resting State BOLD Variability of the Posterior Medial Temporal Lobe Correlates with Cognitive Performance in Older Adults with and without Risk for Cognitive Decline”

**DOI:** 10.1523/ENEURO.0024-22.2022

**Published:** 2022-02-10

In the article, “Resting State BOLD Variability of the Posterior Medial Temporal Lobe Correlates with Cognitive Performance in Older Adults with and without Risk for Cognitive Decline,” by Tyler J. Good, Joshua Villafuerte, Jennifer D. Ryan, Cheryl L. Grady, and Morgan D. Barense, which published online on March 19, 2020, Figure 3*B* appeared incorrectly. The values for “3. Memory” and “4. Intelligence” were inadvertently switched. This error does not affect the conclusion of the results.

**Figure 3.**
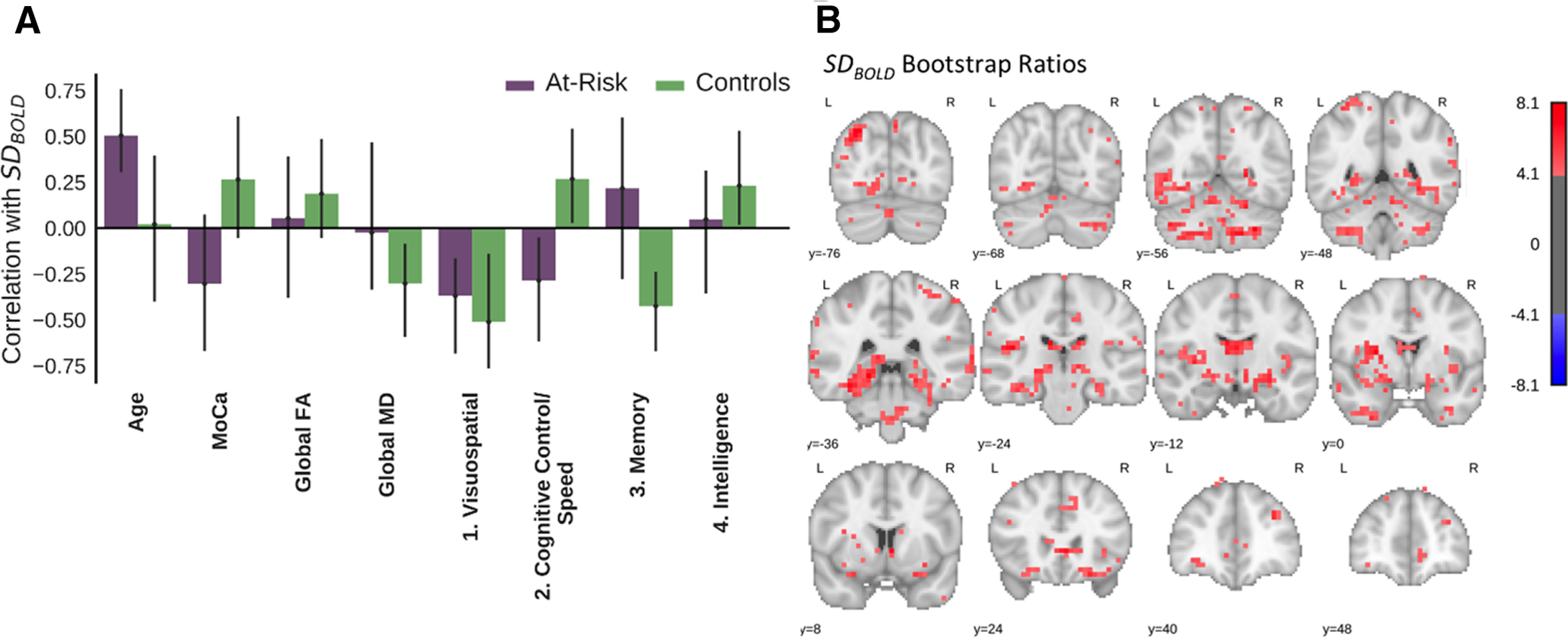
Multivariate PLS analysis of the relationship between SD_BOLD_ and 8 variables (age, MoCA score, global FA and MD, and score on 4 neuropsychological factors). In controls, SD_BOLD_ (particularly in the MTL) was associated with higher cognitive control/speed and intelligence scores, but lower visuospatial and memory scores. These relationships were weaker in the at-risk group. ***A***, The first latent variable (*p *<* *0.001, 41.1% covariance) from the omnibus, between groups behavioral PLS assessing correlations between the 8 variables and SD_BOLD_. The bars represent the correlation between each variable with the pattern of SD_BOLD_ shown in the corresponding brain plot (***B***). The error bars represent 95% confidence intervals, so the error bars of variables significantly contributing to the latent variable will not cross zero. ***B***, Brain plots showing the bootstrap ratios for the latent variable, which may be interpreted like *z* scores. That is, the highlighted voxels are reliably associated with the related variables in ***A*** that significantly contribute to the latent variable. To clearly show the spatial pattern of the respective latent variable, only voxels with bootstrap ratios >|4| are pictured.

