# Supplementary material for: Resting State BOLD Variability of the Posterior Medial Temporal Lobe Correlates with Cognitive Performance in Older Adults with and without Risk for Cognitive Decline
Source: eNeuro. 2020 May 20;7(3):ENEURO.0290-19.2020. doi: 10.1523/ENEURO.0290-19.2020 (PMC7240288; doi:10.1523/ENEURO.0290-19.2020)
Supplement: Table 2-2 — Supplementary Table 2-2. Download Table 2-2, DOCX file. [file enu-eN-NWR-0290-19-s02.docx]

**Table 2-2**





(Olsen et al., 2017)

*the provided significance values are uncorrected for multiple comparisons
